# Endogenous adaptation to low oxygen modulates T-cell regulatory pathways in EAE

**DOI:** 10.1186/s12974-015-0407-4

**Published:** 2016-01-19

**Authors:** Nilufer Esen, Vladimir Katyshev, Zakhar Serkin, Svetlana Katysheva, Paula Dore-Duffy

**Affiliations:** Department of Neurology, School of Medicine, Wayne State University, Detroit, MI 48201 USA

**Keywords:** Multiple sclerosis, EAE, Hypoxia, Adaptation, Angioplasty, Microvessels, HIF-1α, IL-17, T-regulatory cells

## Abstract

**Background:**

In the brain, chronic inflammatory activity may lead to compromised delivery of oxygen and glucose suggesting that therapeutic approaches aimed at restoring metabolic balance may be useful. In vivo exposure to chronic mild normobaric hypoxia (10 % oxygen) leads to a number of endogenous adaptations that includes vascular remodeling (angioplasticity). Angioplasticity promotes tissue survival. We have previously shown that induction of adaptive angioplasticity modulates the disease pattern in myelin oligodendrocyte glycoprotein (MOG)-induced experimental autoimmune encephalomyelitis (EAE). In the present study, we define mechanisms by which adaptation to low oxygen functionally ameliorates the signs and symptoms of EAE and for the first time show that tissue hypoxia may fundamentally alter neurodegenerative disease.

**Methods:**

C57BL/6 mice were immunized with MOG, and some of them were kept in the hypoxia chambers (day 0) and exposed to 10 % oxygen for 3 weeks, while the others were kept at normoxic environment. Sham-immunized controls were included in both hypoxic and normoxic groups. Animals were sacrificed at pre-clinical and peak disease periods for tissue collection and analysis.

**Results:**

Exposure to mild hypoxia decreased histological evidence of inflammation. Decreased numbers of cluster of differentiation (CD)4+ T cells were found in the hypoxic spinal cords associated with a delayed Th17-specific cytokine response. Hypoxia-induced changes did not alter the sensitization of peripheral T cells to the MOG peptide. Exposure to mild hypoxia induced significant increases in anti-inflammatory IL-10 levels and an increase in the number of spinal cord CD25+FoxP3+ T-regulatory cells.

**Conclusions:**

Acclimatization to mild hypoxia incites a number of endogenous adaptations that induces an anti-inflammatory milieu. Further understanding of these mechanisms system may pinpoint possible new therapeutic targets to treat neurodegenerative disease.

## Background

Multiple sclerosis (MS) is a chronic progressive inflammatory demyelinating disease of the CNS. Pathological studies using autopsy material from patients with MS demonstrate the presence of inflammatory cells and their products in the characteristic brain lesions (plaques) associated with this disease. These and other data generated from animal models of MS have led to the generally accepted hypothesis that the disease is mediated by pathogenic leukocyte responses directed against myelin antigens leading to chronic inflammation and ultimately to a broader neurodegenerative process [1–4]. At the vascular level, migrating inflammatory cells release cytokines thought to induce endothelial activation which further promotes leukocyte migration [5, 6]. With continued recruitment and infiltration of increased numbers of leukocytes, there is disruption and dissolution of the intact endothelium [6–8]. Loss of endothelial function disrupts the supply of oxygen and glucose to the nearby tissue promoting metabolic stress [9–11]. Bioenergetic imbalance at the vascular level results in a loss of tissue homeostasis. It has been suggested that changes in metabolic homeostasis in MS may share some similarity to that observed in hypoxia/ischemia and has been described as a “virtual hypoxia” [12, 13]. Thus, therapeutic intervention aimed at the restoration of vascular and metabolic homeostasis should be beneficial in the treatment of MS.

In the brain, maintenance of metabolic homeostasis is the result of a coordinated effort between the cellular constituents of the neurovascular unit (pericytes, endothelial cells, astrocytes, and neurons) [14–16]. These cells make fine-tuned regulatory adjustments that promote survival and maintain the balance between oxygen and glucose availability and neuronal metabolic demand. In response to stress, the brain initiates a number of endogenous adaptive mechanisms that result in a continuous matching of tissue oxygen with capillary density. Adaptive angioplasticity is induced by exercise conditioning [17–19], memory, and learning [20, 21] and upon exposure to mild 10 % oxygen stress [14, 22]. We have extensively studied endogenous adaptation to mild oxygen stress [6, 23, 24]. Exposure to 10 % oxygen is comparable to that encountered at moderate high altitude. Endogenous adaptation to low oxygen is in essence an acclimatization to mild hypoxic stress that promotes tissue survival. We have shown that acclimatization to mild hypoxia ameliorates the signs and symptoms of myelin oligodendrocyte glycoprotein (MOG)-peptide-induced experimental autoimmune encephalomyelitis (EAE) [25]. Exposure of animals to chronic mild hypoxia on the same day of immunization significantly delayed the onset and severity of clinical symptoms. When animals were subjected to chronic mild hypoxia following the appearance of clinical symptoms, chronic inflammation was suppressed [25]. Histological evidence of decreased infiltration of leukocytes was observed in both protocols. Hypoxia-inducible factor-1alpha (HIF-1α) was induced in the spinal cord in EAE associated with infiltrating leukocytes and in vascular pericytes in hypoxia-treated animals. However, the exact mechanism responsible for the therapeutic efficacy in EAE is not yet known.

In the present study, we have investigated the potential mechanisms involved in the amelioration of EAE. We have questioned whether exposure to chronic mild hypoxia altered immune mechanisms responsible for induction and/or perpetuation of disease activity in MOG-peptide-induced EAE. Hypoxia significantly delayed the onset of the signs and symptoms of EAE associated with decreased histological evidence of inflammatory activity in the spinal cord. Decreased evidence of leukocyte infiltration was not due to delayed peripheral sensitization of MOG-reactive lymphocytes as splenocytes isolated from MOG-immunized mice exposed to hypoxia exhibited a similar MOG-induced proliferative capacity as splenocytes from EAE animals exposed to normal levels of oxygen. Amelioration of disease did, however, correlate with changes in the cytokine-secreting phenotype of the T-cell subsets. Decreased infiltration of cluster of differentiation (CD)4+ cells along with the increased number of regulatory CD25+FoxP3+ T cells found in the hypoxia-exposed mice suggest a possible mechanism for the therapeutic potential of adaptive chronic mild hypoxia in EAE.

## Materials and methods

### Immunization protocol for MOG-induced EAE

All animal experiments were approved by the Institutional Animal Care and Use Committee, Wayne State University (WSU), and conducted in accordance with the WSU guidelines (protocol # A-09-11-12). Female C57BL/6 mice (6–8 weeks, cat # 000664) purchased from Jackson laboratories (Bar Harbor, MN) were immunized with myelin oligodendrocyte glycoprotein (MOG) peptide 35–55, (MEVGWYRSPFSRVVHLYRNGK) (100 μl) (final concentration = 200 μg) emulsified with complete Freund’s adjuvant (CFA) containing 5 mg/ml mycobacterium tuberculosis (Lot # 8183887, Difco Laboratories, Detroit, MI) subcutaneously in two injection sites at the hind flanks of each mouse. Control animals were immunized with an equivalent volume of CFA emulsion that did not contain MOG peptide. All mice received an intraperitoneal (ip) injection with 150 μl of pertussis toxin (300 ng) (List Biological Laboratories, Campbell, CA) immediately after immunization and again 2 days later. Mice were evaluated on a daily basis for changes in body weight, overt signs of illness, and clinical signs of EAE using a five-point scoring system: 0—no symptoms; 1—limp tail; 2—limp tail and hind limb weakness; 3—partial hind limb paralysis; 4—full hind limb paralysis; 5—moribund state or death by EAE. Daily measurements of weight and hematocrit were also made.

### Normobaric hypoxia chamber

Hypoxia chambers (Biospherix, Redfield, NY) can be calibrated to create an environment from 0 to 95 % oxygen. There are two mechanisms for maintaining low-oxygen content (8–15 %): (1) selective oxygen vents along the sides can be stopped up or unplugged to allow the free flow of oxygen into the chamber (normoxic controls) and (2) the ProOx controller monitors/feeds nitrogen into the chamber and responds to the current flow of oxygen. This controller calibrates the levels of oxygen and nitrogen using feedback algorithms. Oxygen-sensing equipment was used to verify the concentrations indicated on the controller.

### Exposure to normobaric hypoxia

Animals are housed in the Biospherix hypoxia chambers calibrated to administer 10 % oxygen for varying periods of time. In this study, mice were exposed to hypoxia at day 0 (day of immunization). Control normoxic animals were house on the bench top next to the hypoxia chambers under normal oxygen conditions. Following exposure to moderate low oxygen, animals were sacrificed for tissue acquisition at varying times per our approved protocol.

### Histology, vascular density, and diaminobenzidine (DAB) staining

Frozen sections (5 μm) were fixed with 4 % paraformaldehyde, permeabilized with 0.1 % Triton X-100, quenched with 0.3 % H_2_O_2_ (in 30 % methanol), and incubated with 5 % normal rabbit serum. The sections were incubated with goat polyclonal anti-glucose transporter protein-1 (Glut-1) Ab (1:100, Santa Cruz Biotechnology, CA) and a biotinylated secondary antibody (Vector Laboratories, CA) for vascular density. Color detection was performed with avidin-biotin horseradish peroxidase solution, ABC kit, and the diaminobenzidine (DAB) peroxidase substrate kit (Vector Laboratories, CA, USA). Sections were prepared at random from a minimum of four samples of the spinal cord. Images were captured using a Pentax K10D camera mounted to an Axiovert 135 light microscope. For vascular density, the number of vessels per field was determined on 20–30 fields per sample from a randomly selected section of the lumbar spinal cord and presented as the mean ± SD.

### Immunocytochemistry

Experimental animals were perfused with saline and 4 % paraformaldehyde, and the brain was immersed in the same fixative for 1–2 days. After fixation, the spinal cords were dissected in 1-cm^3^ blocks, cyroprotected with sucrose, and frozen on dry ice. Sections, 8 μm thick, were prepared with a cryostat (Carl Zeiss, Microm HM505N, Germany). The sections were stored at −20 °C before use. The series consisted of tissue from each group of animals stained with a rabbit anti-mouse HIF-1α IgG2 (Santa Cruz, Santa Cruz, CA) followed by a goat anti-rabbit IgG-FITC-conjugated (Jackson Immunoresearch, West Grove, PA, USA) secondary antibody used at saturation density. When appropriate, DAPI (Molecular Probes, Eugene, OR) was used to visualize the nucleus. Control sections omitting primary antibodies were used to assess nonspecific binding. Some sections were also stained with hematoxylin and eosin (H&E) to visualize infiltrating cells.

### Assessment of tissue hypoxia

The assay is based on the chemical reaction of injected pimonidazole which is activated in hypoxic cells and forms stable adducts with thiol groups in proteins, peptides, and amino acids. Hypoxic cells bind pimonidazole (2-nitroimidazoles) to peptide thiols such as glutathione. Although it is difficult to measure Km (O_2_) for 2-nitroimidazole binding in solid tissue, it has been shown that 10 mmHg is a reasonable threshold value for pimonidazole binding in solid tissue. Sham- or MOG-immunized mice either exposed to 10 % O_2_ or 21 % normoxia were injected with 50 mg/kg pimonidazole and 5 mg/kg Hoechst dye mixture i.p. and perfused with PBS and 4 % PFA 30 min later. Cryostat sections were stained following the manufacturer’s instructions (Hypoxyprobe, Inc., MA).

### Western analysis

The expression of HIF-1α in the spinal cords of mice was evaluated by Western blot analysis. Protein extracts were prepared by lysing tissue homogenates in 500 μl of RIPA buffer [1 % NP-40, 0.1 % sodium dodecyl sulfate (SDS) in phosphate-buffered saline (PBS), pH 7.4] supplemented with a Complete^TM^ protease inhibitor cocktail tablet (Roche, Indianapolis, IN, USA). Lysates were allowed to incubate on ice for 30 min followed by centrifugation at 13,000 rpm for 15 min at 4 °C to pellet debris. Calculation of total protein in microglial extracts was determined by using a standard protein assay [bicinchoninic acid (BCA) protein assay reagent; Pierce, Rockford, IL, USA]. Protein extracts (40 μg of the total protein) were electrophoresed on 4–10 % Tris–HCl Ready Gels (Bio-Rad, Hercules, CA, USA) and transferred to a polyvinylidene difluoride (PVDF) membrane (Immobilon-P, Millipore, Bedford, MA, USA) using a wet transfer apparatus (Bio-Rad). Blots were probed using a rabbit anti-mouse HIF-1α (Santa Cruz) followed by a donkey anti-rabbit IgG–HRP conjugate (Jackson Immunoresearch, West Grove, PA, USA). Blots were stripped and re-probed with a mouse anti-actin monoclonal antibody (Santa Cruz) to verify uniformity in gel loading. Blots were developed using the ChemiGlow West substrate (Alpha Innotech, San Leandro, CA, USA) and visualized by exposure to X-ray film (Kodak Biomax, Rochester, NY, USA).

### Isolation of spinal cord leukocytes and flow cytometry

To determine whether exposing immunized mice to hypoxia influenced the influx of immune cells, T cells, polymorphonuclear leukocytes (PMNs), macrophages, and microglia were quantitated by fluorescence-activated cell sorting (FACS) analysis, as previously described [26]. Briefly, mice were perfused to eliminate leukocytes from the vasculature, whereupon the brain and spinal cord were collected to recover infiltrating cells. Following vascular perfusion, tissues were minced in HBSS (HyClone, S. Logan, UT) and digested filtered through a 70-μm nylon mesh cell strainer using a rubber policeman. The resulting slurry was digested for 30 min at 37 °C in HBSS supplemented with 0.2 mg/ml collagenase type I (Sigma-Aldrich, St Luis, MO), 5000 U/ml DNase I (Invitrogen, NY), 1 mg/ml dispase (Invitrogen), and 0.025 % trypsin to obtain a single-cell suspension. Following enzyme neutralization, cells were layered onto a discontinuous OptiPrep (Fisher) gradient and centrifuged at 2000 rpm for 15 min at room temperature in a swinging bucket rotor. After centrifugation, myelin debris was carefully aspirated and the cell interface was collected. Following extensive washes and incubation in Fc block (BD Biosciences, CA) to minimize nonspecific Ab binding to FcRs, cells were stained with directly conjugated Abs for the four-color FACS to detect PMNs and macrophages (CD11b+, CD45^high^) and microglia (CD11b+, CD45^low-intermediate^). To enumerate EAE-associated T-cell infiltrates, cells were analyzed using antibodies to CD3, CD4, and CD25. For the FoxP3, staining cells were fixed with 4 % paraformaldehyde and permeabilized with 0.1 % saponin before incubating with the antibody. All Abs were purchased from BD Biosciences. Cells were analyzed using a BD FACSAria with the compensation set based on the staining of each individual fluorochrome alone and correction for autofluorescence with unstained cells.

### Isolation of splenocytes

Spleens of both hypoxia-exposed and normoxic EAE mice were removed and minced in PBS having 2 % FBS. Then they passed through a 70-μm cell strainer, washed and then centrifuged. The pellet is resuspended and red blood cells lysed with ACK lysing buffer. After the red cell lysis cells are washed and suspended in Dulbecco’s modified Eagle’s medium (DMEM), cell suspensions are kept in complete DMEM medium on ice.

### T-cell stimulation

Single-cell suspensions of splenocytes were prepared from animals exposed to normoxia (immunized only) or to hypoxia (immunized plus low oxygen) as well as from naïve mice. The cells were plated into 96-well plates at a concentration of 1 × 10^5^ cells/well and incubated with MOG peptide (50 μg/ml) and nonspecific mitogen concanavalin A (ConA). Cultures were maintained at 37 °C in humidified 5 % CO_2_/air. Cells were pulsed with 1 mCi [3H]-thymidine after 72 h and then harvested 18 h later on a Filtermat for scintillation counting. The results were determined as means from triplicate cultures and are shown with SEM.

### IFN-γ and IL-17A ELISPOT assay

The MOG-specific response of T cells isolated from immunized mice either exposed to hypoxia or normoxia was analyzed for cytokine-secreting phenotype by ELISPOT assay. Briefly, splenocytes (2 × 10^5^ cells/well) were plated into IL-17A or IFN-γ-coated 96-well filtration plates (Millipore) and stimulated with MOG peptide overnight at 37 °C in humidified 5 % CO_2_/air incubators. After 24 h, the plates were incubated with the appropriate biotinylated antibodies (all antibodies from ebiosciences), and the cytokine-producing cells were visualized with streptavidin-alkaline phosphatase system (Vector Laboratories), and the plates were analyzed using the CTL ImmunoSpot Analyzer (Cellular Technology Limited, Shaker Heights, OH) with ImmunoSpot software. The frequency of cytokine-producing cells was expressed as the difference between the mean number of spots and the mean background for each experiment. The data were presented as the mean ± SEM of each group, *n* = 3 mice, and performed in triplicate.

### Statistics

All histological and immunocytochemical analyses are conducted using four to six sections per animal with three to six areas of analysis per section and four to six animals per group (see the “Materials and methods” section). Data is expressed as an average of each area of analysis. Between-group analysis was accomplished using one-way analysis of variance (ANOVA) with least significant difference post hoc testing. Significance is set at *p* value <0.05. Based on the variability in these data from our previous studies [25], we can distinguish a difference in individual proteins and in capillary density with 95 % power.

## Results

### Endogenous adaptation to chronic mild hypoxia

**Fig. 1:**
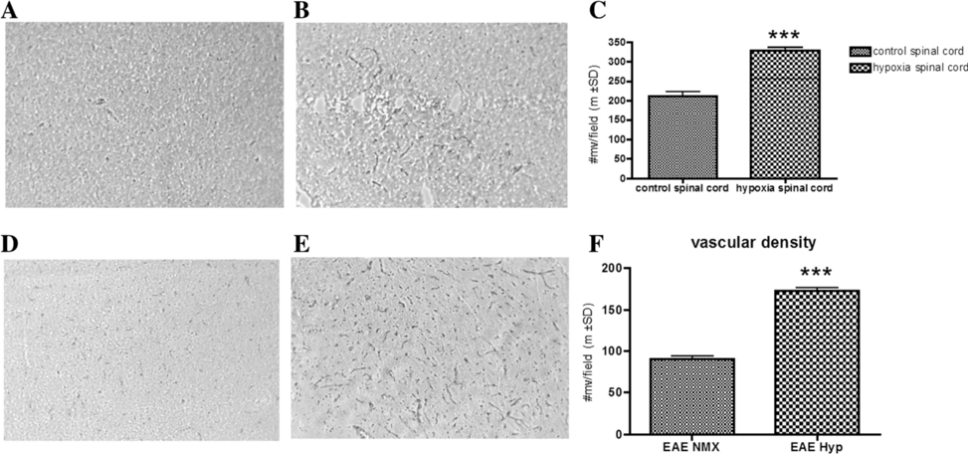
Hypoxia resulted in increased capillary density. Spinal cord sections of mice exposed to 10 % hypoxia for 3 weeks and normoxic controls were prepared as detailed in the “Materials and methods” section. Glut-1+ microvessels were counted in 20–30 fields per sample from normoxia- (**a**) and hypoxia- (**b**) exposed mice. The *bar graphs* (**c**) are the mean ± SD number of vessels per field. Glut-1+ microvessels were also more in MOG-immunized hypoxia-exposed spinal cords (**e**), when compared to normoxic MOG-immunized spinal cords (**d**). Quantification of those microvessels was presented as *bar graph* (**f**) (mean ± SD of at least three mice per group). Significant differences between the groups are denoted with *asterisks* (*** *p* < 0.001)

**Fig. 2:**
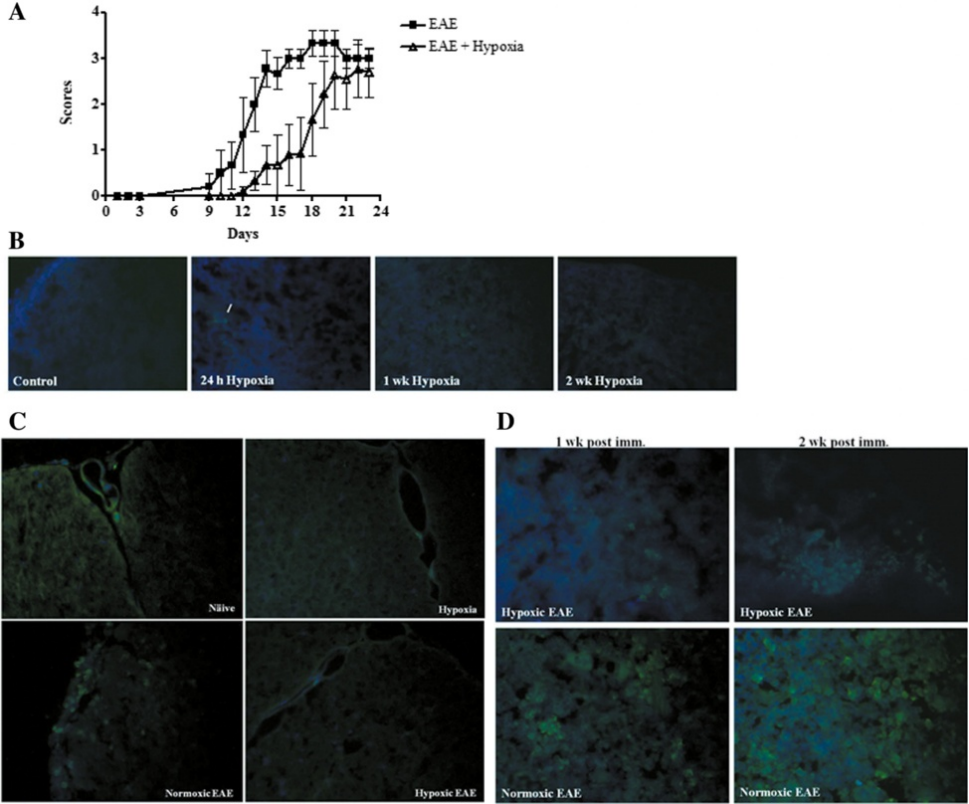
Chronic mild hypoxia delayed onset of EAE and caused less tissue hypoxia. Mice were immunized with MOG35-55 peptide+ CFA. Immediately following immunization, one group of animals was placed in the normobaric hypoxia chambers. The EAE-only group was left in the normoxic environment all the time. Clinical scores were determined daily. **a** The graph presents the mean ± SD of scores (*n* = 12 mice/group) including the score 0 from two independent experiments. Results confirm previous studies using smaller numbers of animals. The two-way ANOVA analysis revealed that the difference was statistically significant (*p* < 0.001). Tissue hypoxia was determined in spleens and spinal cords of all animals with pimonidazole (*green*) and Hoechst (*blue*, for nuclei) as described in the “Materials and methods” section. **b** Spleens of control and hypoxic mice at different time points following 10 % O_2_ treatment are representative of three mice/group/time point. Few positive cells (*arrow*) were detected only at the 24-h post-hypoxia group. Spinal cords at the peak disease (**c**) and spleens (**d**) after 1 and 2 weeks of EAE induction (*n* = 3/group). Magnification is ×40

In vivo exposure to chronic mild hypoxia (10 % oxygen) under normobaric conditions induces a set of well-characterized adaptive changes [14, 22] that in the CNS includes increased vascular density. Evidence of systemic adaptation to the stress stimuli is observed as early as 4–6 h and structural evidence by 7 days. By 3 weeks, there is a near doubling of vascular density (Fig. 1a–c). The adaptive response to hypoxia is protective and important to tissue survival and plasticity. In our first publication, we questioned whether mild oxygen stress altered EAE [25]. We found that induction of the stress response was protective (28). In this paper, we further define the mechanism of action. MOG-immunized and sham-immunized mice were housed at 10 % oxygen in normobaric hypoxia chambers or on the bench top next to the chambers for 0–21 days from the day of immunization. Animals were scored daily for evidence of clinical disease using a five-point scoring scale. Animals were weighed, and blood samples taken for hematocrit. Spinal cord and brain tissue samples were obtained on 0, 7, 14, and 21 days post-immunization. Vascular density in the spinal cord was determined in immunized mice exposed to normoxic conditions and immunized animals exposed to 10 % low oxygen. Results confirmed previous studies [25] and indicated that vascular density is somewhat reduced in immunized normoxic mice when compared to sham-immunized animals. Exposure to mild hypoxia in treated immunized animals resulted in increased vascular density, but the total numbers of vessels per field was less than sham-immunized hypoxia-treated animals (Fig. 1d–f). Exposure to hypoxia on the same day as immunization induced a statistically significant (*p* < 0.01) delay of disease onset (Fig. 2a). Hypoxia-treated immunized animals ultimately developed disease although the severity of diseases was somewhat variable. Disease incidence was similar (80 to 90 %) in both immunized animal groups.

### Tissue hypoxia following adaptation to chronic mild hypoxia

We questioned whether changes in endogenous tissue hypoxia may contribute to changes in EAE pathogenesis. To answer this question, we evaluated tissue hypoxia following administration of a novel hypoxia marker pimonidazole [27]. Pimonidazole binds to thiol-containing proteins specifically in hypoxic cells. Hypoxic cells can be visualized and recognized by immunohistochemical detection of pimonidazole using a mouse monoclonal antibody. Pimonidazole was injected at the indicated time points and tissue harvested. When animals were exposed to hypoxia for 24 h, we found very few pimonidazole-positive cells after. Levels were comparable to normoxic animals. After continued exposure to low oxygen for 1 or 2 weeks, the number of pimonidazole-positive cells decreased (Fig. 2b). In EAE-induced animals, pimonidazole staining was detectable in splenocytes and infiltrating cells of spinal cord sections at the peak of disease (Fig. 2c, d) indicating that induction of the inflammatory response increased evidence of tissue hypoxia. Exposure of EAE animals to 10 % oxygen decreased the number of positively staining cells (Fig. 2c, d). Reduced tissue hypoxia correlated with increased vascular density. These results indicate that in vivo exposure to chronic mild hypoxia does not further increase tissue hypoxia. Rather, they suggest that adaptation to a mild hypoxic stress dampens the intensity of pimonidazole staining in treated animals.

### Endogenous adaptation to chronic low oxygen and amelioration of EAE is mediated by HIF-1α

**Fig. 3:**
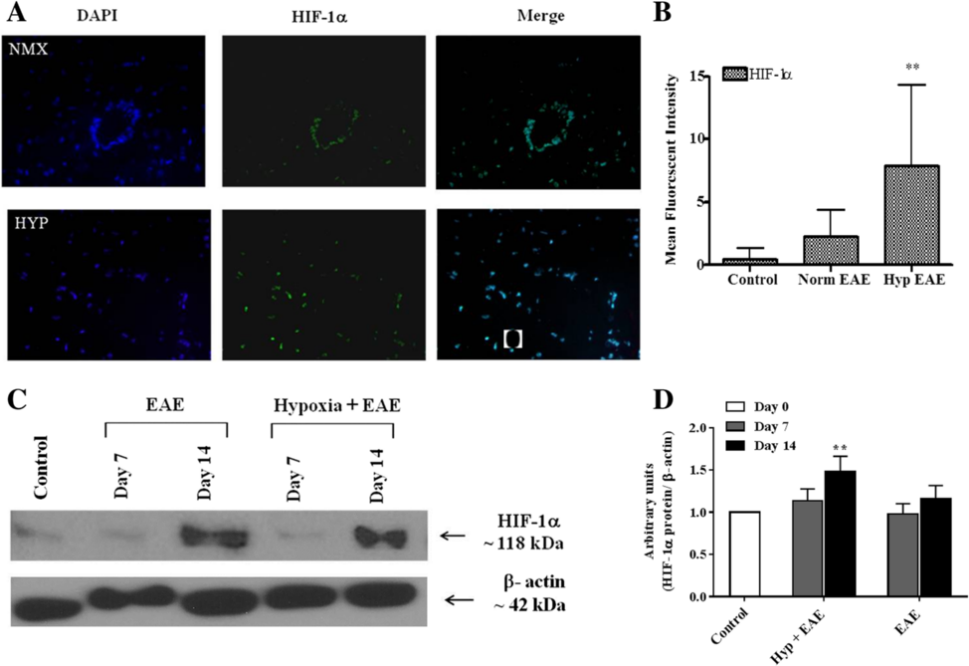
HIF-1α expression was induced both in hypoxia-treated and normoxic EAE animals. **a** Flash-frozen spinal cord sections of both hypoxic and normoxic EAE mice (*n* = 3/group) at peak disease were prepared as mentioned in the “Materials and methods” section and subjected to immunofluorescence staining for HIF-1α (*green*) and DAPI (*blue*, for nuclei). To show the distribution differences, pictures of ×10 magnification were presented. Results are representative of two independent experiments. **b** Mean fluorescent intensity of HIF-1α-stained sections were quantified using ImageJ program, and the *graphs* represent the mean of fluorescent intensity from 10 to 14 areas per group (mean ± SD). Significant differences between hypoxia-exposed and normoxic EAE mice are denoted with *asterisks* (**p < 0.01). **c** Spinal cord protein extracts were prepared at the indicated days post-immunization and analyzed for HIF-1α expression by Western blotting as described in the “Materials and methods” section. To compare the baseline expression of HIF-1α, naïve spinal cord was also included. Membranes were stripped and re-probed with β-actin. Results are representative of three samples/time point/group. **d** WB films (*n* = 3/group) were scanned, and the intensity of bands was quantified using ImageJ program. The *graph* shows the relative expression of HIF-1α in the groups following normalization first to its β-actin band and then to the control group. *Asterisks* denotes the significant differences between hypoxia-exposed EAE mice and the control mice (**p < 0.01)

Endogenous adaptation to low oxygen is mediated by HIF-1α. In the hypobaric model in rats, hypoxia-induced HIF-1 expression is first seen in pericytes (4–6 h) and subsequently in glial cell populations by 24–48 h. Increased vascular density is associated with up-regulation of both HIF-1α-dependent and HIF-1α-independent signaling molecules. Similar results are observed in the normobaric hypoxia model used in these studies. Following MOG immunization under normoxic conditions, HIF-1α is predominantly expressed in infiltrating leukocytes located in perivascular cuffs (Fig. 3b). In hypoxia-treated MOG-immunized animals, HIF-1α expression in the spinal cord was dispersed in multiple cell populations (Fig. 3a, b). Induction levels were significantly higher in the spinal cords of mice exposed to hypoxia during the later time points of disease when compared to sham-immunized mice. However, image analysis and arbitrary unit results did not reach statistical significance when HIF-1α expression of hypoxia-exposed EAE mice was compared to that of normoxic EAE mice (Fig. 3c, d).

### Exposure to mild hypoxia modulates immune activity in EAE

**Fig. 4:**
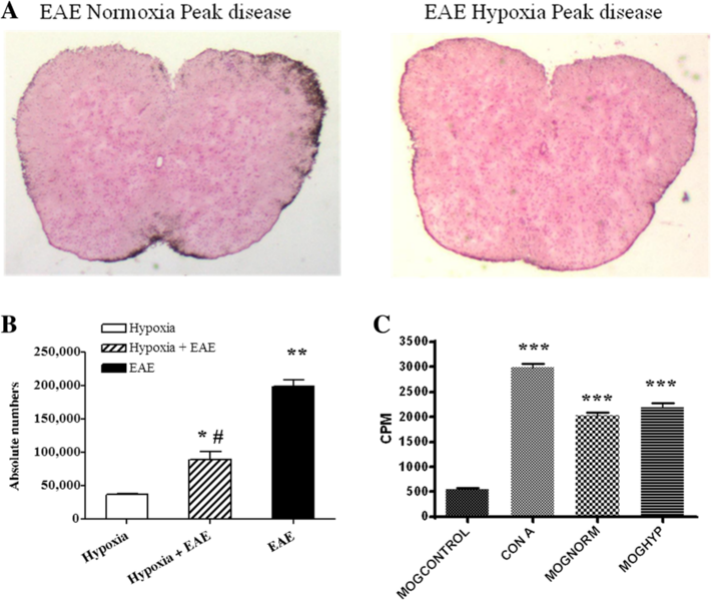
Hypoxia treatment attenuated the inflammation in EAE. Mice were immunized with MOG peptide and housed in hypoxic chambers at day 0. Spinal cord sections were prepared at the peak disease and stained with H&E to visualize infiltrating cells (×2) (**a**). Results are representative of three mice per group and repeated at least twice. **b** Total numbers of myeloid cells were isolated from the spinal cords at peak disease as described in the “Materials and methods” section. Significant differences when compared to control mice are denoted with *asterisks* (*p < 0.05, **p < 0.01, ***p < 0.001), while the *pound sign* denotes the significant differences between hypoxia-exposed and normoxic EAE mice (^#^ *p* < 0.05). However, the MOG-induced proliferative capacity of splenocytes was not affected by mild hypoxia, when determined through [3H]-thymidine incorporation and counted by using a scintillation beta-counter as mentioned in the “Materials and methods” section (**c**). The results are presented as the mean ± SD of cpm values from triplicate cultures. Significant differences between the control group and the stimulated cells are denoted with *asterisks* (***p < 0.001)

As noted above, MOG-immunized mice exposed to mild low oxygen exhibited delayed onset of the signs and symptoms of EAE. When we examined the spinal cord for histopathological evidence of disease, we found that exposure to mild low oxygen decreased histological evidence of inflammation (Fig. 4a). Both the number of visible leukocyte plaques and the total number of myeloid cells that could be isolated from the spinal cords were significantly decreased in hypoxia-treated EAE mice (Figs. 4b, and 7b).

**Fig. 5:**
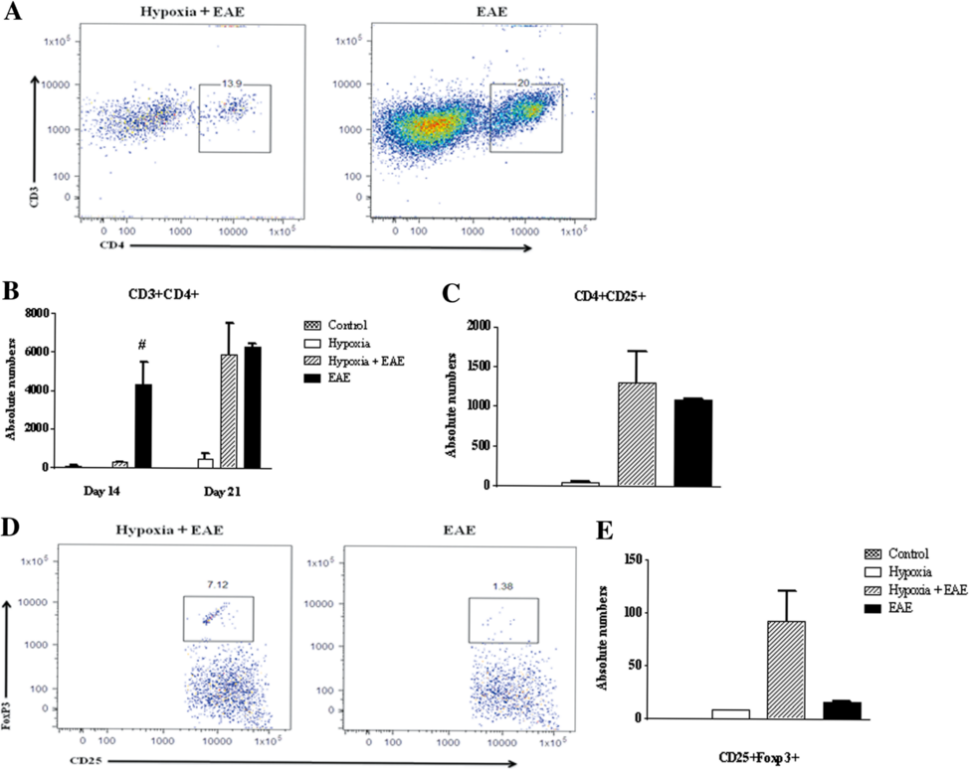
CD4+ T cells were less in the hypoxic EAE spinal cords at day 14. Immune cells were recovered from mice (three animals/group/time point) at days 7 and 14 following MOG immunization as described in the “Materials and methods” and analyzed by FACS. Gating was done first on the CD11b-CD45hi cells and then further plotted for CD3, CD4, and CD25 expressions (**a**). Total numbers of CD3+CD4+ (**b**) and CD4+CD25+ (**c**) cells in the spinal cords were presented (mean ± SD). A representative *dot plot* of CD4+CD25+FoxP3+ (**d**) and the absolute numbers (**e**) are presented (mean ± SD). Significant differences between hypoxia-exposed and normoxic EAE mice are denoted with *pound sign* (^#^ *p* < 0.05)

Decreased leukocyte infiltration into the spinal cord may reflect less than optimal peripheral sensitization of T cells. We, therefore, questioned whether exposure to low oxygen altered the activation level of T cells. Evidence of antigen-induced sensitization of T cells was determined by measuring the proliferative capacity of splenocytes to the encephalitogenic peptide MOG35-55 compared to a nonspecific mitogen concanavalin A (ConA). As seen in Fig. 4c, splenocytes isolated from MOG-immunized mice exposed to hypoxia exhibited similar MOG-induced proliferative capacity as splenocytes from EAE animals exposed to normal levels of oxygen. No differences were seen in response to ConA. Thus, hypoxia did not influence MOG-specific T-cell sensitization and proliferation in response to specific antigen or mitogen challenge (Fig. 4c). We then questioned whether low oxygen altered infiltrating T cells. Activated T cells were detectable at days 10–14 post-immunization in both hypoxic and normoxic MOG-immunized EAE mice. However, the percentage of CD4+ T cells was significantly less in the spinal cords of hypoxia-exposed EAE mice (Fig. 5a, b). While the presence of CD4+ cells in the spinal cord is highly correlated to disease severity [28], the presence of regulatory T cells [CD25+FoxP3+ regulatory T cells (Tregs)] can dampen neuroinflammation within the CNS. Tregs have been shown to dampen JHMV-infected mice [29]. In our studies, we found increased numbers of FoxP3 expressing CD25+ T cells in the hypoxic EAE spinal cords (Fig. 5c–e).

#### Activation of IL-17-secreting T cells is delayed in animals exposed to low oxygen

**Fig. 6:**
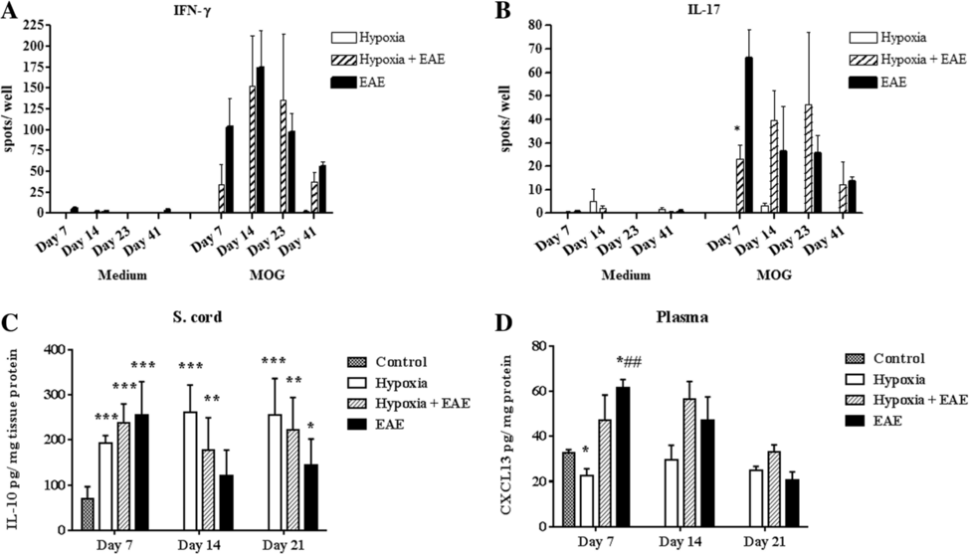
Hypoxia delayed the sensitization of IL-17A-producing T cells and modulated the inflammatory mediators. Spleens were removed from either hypoxic or normoxic MOG-immunized or control mice at days 7, 14, 23, and 41 post-immunization, and single-cell suspensions (at 2 × 10^5^ cells/well) were plated into either IFN-γ- (**a**) or IL-17A- (**b**) coated 96-well filtration plates in the presence of MOG or medium alone. Following the overnight incubation, spots in the wells were analyzed using CTL ImmunoSpot Analyzer and the software. The data were presented as the mean ± SEM of each group, *n* = 3 mice, and performed in triplicate. Significant differences between hypoxia-exposed and normoxic EAE splenocytes are denoted with *asterisks* (*p < 0.05). IL-10 (**c**) and CXCL13 (**d**) levels in the spinal cord tissue homogenates were determined following the manufacturer’s instructions. Significant differences when compared to control mice are denoted with *asterisks* (*p < 0.05, **p < 0.01, ***p < 0.001), while the *pound sign* denotes the significant differences between hypoxia-exposed and normoxic EAE mice (^##^ *p < 0.01*)

We also determined the T-cell cytokine-secreting phenotype as a means of pinpointing the functional activity of the T cells. To visualize the T helper (Th)1 responses, we examined IFN-γ production in both hypoxic and normoxic EAE mice. When compared to hypoxia only and naïve mice, IFN-γ was significantly induced in both hypoxic and normoxic EAE mice and reached a peak at day 14 (Fig. 6a). The difference between IFN-γ production by hypoxic versus normoxic cells was statistically significantly different at all time points examined. On the other hand, Th17-specific cytokine IL-17A production was significantly less in cells derived from EAE mice exposed to low oxygen than was determined for cells isolated from untreated EAE animals (Fig. 6b). At later time points, no significant differences were seen. These data suggest that the activation of Th17 cells, which are claimed to be encephalitogenic and play a major role in disease pathogenesis, is delayed when animals are exposed to hypoxia at the time of immunization. This may be directly relevant to the late onset of disease observed in previously discussed experiments.

#### Hypoxia modulates CNS levels of IL-10 and CXCL13

Interleukin 10 (IL-10) is one of the most important anti-inflammatory cytokines since it inhibits pro-inflammatory cytokine production by suppressing the expression of MHC-II, adhesion molecules, and co-stimulatory molecules on antigen-presenting cells. It has been shown that IL-10 is produced by T cells [30] and macrophages [31] under hypoxic conditions and promotes angiogenesis [31, 32]. However, contradictory results are presented by the studies that evaluated its role in MS [33, 34] and EAE [35], suggesting a diverse and more complex role of IL-10 in CNS inflammation. In our study, following exposure to the chronic mild hypoxia, the levels of anti-inflammatory cytokine IL-10 was induced in the spinal cords of sham-immunized mice as well as MOG-peptide-immunized mice. Interestingly, IL-10 was maintained at high levels in the sham-immunized mice during all time points investigated, while its levels gradually decreased in both hypoxic and normoxic EAE mice (Fig. 6c). Since IL-10 was induced under both hypoxic and inflammatory conditions but declined in the inflammation while staying high in hypoxic mice over the course we examined, IL-10 production is possibly regulated by multiple and complex factors. On the other hand, it is well established that the chemokine CXCL13 is found at high levels in the serum and cerebrospinal fluid (CSF) of patients with MS [36, 37] and reported to promote EAE [38, 39]. As expected, CXCL13 was found at low levels in the plasma of hypoxia-treated sham-immunized mice at 7 days pi (Fig. 6d). Hypoxia-treated MOG-immunized mice exhibited CXCL13 levels that while higher than sham-immunized mice were significantly lower than untreated EAE animals. Levels ultimately increased in hypoxia-treated immunized mice. Taken together, these results suggest that changes in the cytokine and chemokine repertoire during the early stages of disease may have a profound effect on the disease induction.

### Hypoxia-induced CD11b+CD45^low^ microglia in the CNS of EAE mice

**Fig. 7:**
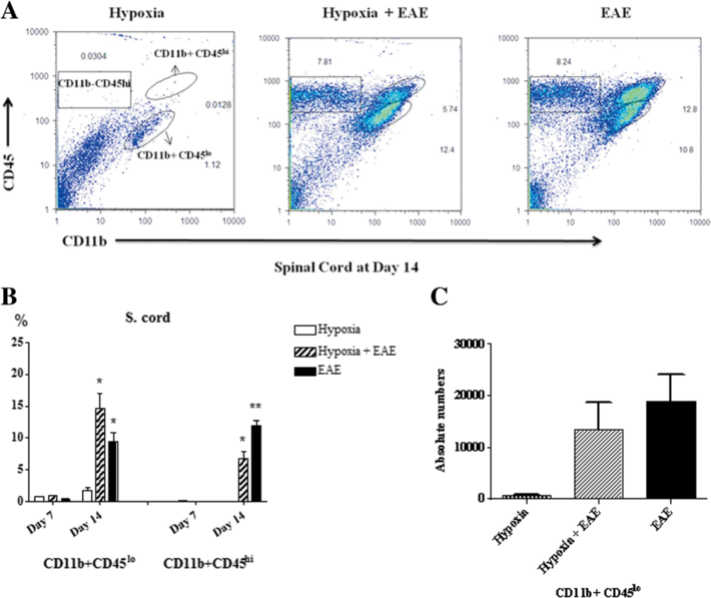
CD11b+CD45^low^ microglial cells are increased in response to mild hypoxia. Immune cells were recovered from the spinal cords (**a**) of mice (three animals/group/time point) at day 14 following MOG immunization as described in the “Materials and methods” section and analyzed by FACS. Results are presented as the percent positive cells for each population (mean ± SD) (**b**) and the absolute numbers of CD11b+CD45^low^ microglia (**c**). Significant differences when compared to hypoxia-only control mice are denoted with *asterisks* (*p < 0.05, **p < 0.01)

Histological examination of spinal cords indicated that exposure to moderate low oxygen decreased the number of infiltrating cells (Fig. 7a, b). The number of microglia, resident antigen-presenting cells of CNS, and infiltrating myeloid cells neutrophils/macrophages in the spinal cords was determined. Cell populations were distinguished by phenotypic expression of cell-specific markers CD11b and CD45. Microglia are CD11b+CD45^low^, while neutrophils and macrophages are CD11b+CD45^high^ expressing cells (Fig. 7a–c). At 7 days post-immunization, CD11b+CD45^high^ expressing cells from both treatment groups could not be detected in spinal cords (Fig. 7b). However, significant numbers were present by day 14 (Fig. 7b) confirming in part the work of other investigators [40, 41]. The percentage of CD11b+CD45^high^ cells that had infiltrated into the spinal cord of hypoxia-treated MOG-immunized mice was significantly less than seen in normoxic EAE mice (Fig. 7b). On the other hand, under hypoxic conditions, percentages of CD11b+CD45^low^ microglial population were increased in hypoxic EAE compared to normoxic EAE, especially at day 14 post-immunization (Fig. 7b, c). Since there were more cells infiltrated in the spinal cords of normoxic EAE mice, the absolute numbers of the CD11b+CD45^low^ microglia population were found not significantly different between the hypoxic and normoxic EAE animals (Fig. 7c). A recent study shows that resident microglia not infiltrating monocytes help to clear the debris in EAE [42]. Therefore, we suggest that and increased number of microglia under hypoxic conditions may have a beneficial effect in EAE pathogenesis. Whether hypoxic conditions also affect the activation level of these cells remains to be determined.

## Discussion

In the brain, metabolic homeostasis is finely tuned and maintained by the cellular constituents of the neurovascular unit (pericytes, endothelial cells, astrocytes, and neurons) [14–16] and is essential for the survival of neurons and the other resident cells of CNS. To achieve balance, endogenous mechanisms have been developed to continuously match oxygen utilization and demand to capillary density. As such, structural adaptations that result in vascular remodeling have been shown to underlie endogenous adaptation to oxygen stress and are thought to promote tissue survival and plasticity [10, 43]. We questioned whether an endogenous adaptive mechanism induced in response to mild stress would alter inflammatory mechanisms in EAE and neurodegenerative disease. In this study, adaptive vascular remodeling was assessed by the up-regulation of hypoxia-responsive elements and evidence of increased vascular density in exposed animals. Both sham-immunized and MOG-immunized animals respond to mild hypoxia associated with an increase in expression of HIF-1α and the HIF-1-dependent genes such as vascular endothelial growth factor (VEGF). VEGF promotes survival of endothelial cells in in vivo and in vitro [44] and induces angioplasticity [44] increasing vascular density. HIF-1α was induced in both MOG-immunized mice, MOG-immunized mice plus hypoxia, and sham-immunized mice plus hypoxia. However, the specific cellular localization was significantly different in the experimental groups. HIF-1 cellular localization in healthy mice exposed to mild hypoxia is associated with pericytes, glial cells, and ultimately neurons. In MOG-immunized animals housed under normoxic conditions, HIF-1α expression was confined to the infiltrating leukocyte populations in plaque areas confirming reports by others [25, 45]. Expression of HIF-1α in immunized animals exposed to low oxygen was associated with a population of cells evenly distributed within the spinal cord. Preliminary evidence suggests that at least a proportion of these cells may be vascular pericytes. Upon exposure to mild hypoxia, pericytes migrate from their microvascular location and proliferate before renewing coverage of newly formed vessels. Pericyte responses to chronic mild hypoxia are discussed in more detail in a review by Dore-Duffy and LaManna [14]. In response to chronic mild hypoxic stress, HIF-1α is stabilized and can in turn bind to hypoxia-responsive elements (HREs) that regulate the transcription of target genes, including those encoding erythropoietin, glucose transporters, glycolytic enzymes, antimicrobial factors, and the angiogenic factor VEGF. Stabilization of HIF-1α is an early event in the adaptive response to low oxygen that leads to a cascade of HIF-1-dependent and HIF-1-independent events that culminates with increased vascular density by 2–3 weeks. The transient expression of signaling molecules in this cascade and that hypoxia did not alter T-cell peripheral sensitization may account for the fact that animals eventually developed disease. HIF-1 levels can also be regulated by a number of signaling pathways important in immune responses as well, notably by those of NFκB and STAT3 [46].

Inflammatory sites can also be characterized as hypoxic milieu due to a focal increase in oxygen demand due to accumulation of activated inflammatory cells [47, 48]. Vascular cuff formation is associated with evidence of BBB damage and undoubtedly compromised vascular homeostasis. Recently, Davies et al. [49] has shown pimonidazole staining in the spinal cords of EAE-induced rats. In our study, we found that pimonidazole staining was restricted to splenocytes and leukocytes infiltrating the spinal cord in MOG-immunized EAE mice. We further show that endogenous adaptation to chronic mild hypoxia reduced pimonidazole staining in EAE mice and produced a clinical benefit in EAE. In the Davis study, induction of hyperoxia did not significantly alter clinical disease in EAE in rats. They interpreted their studies to indicate that hypoxia was detrimental and necessary for the development of disease and suggested that hyperoxia may be beneficial as a therapeutic modality in MS. However, they did not distinguish between endogenous and inflammatory-mediated hypoxia. Hyperoxygenation of hypoxic tissue has been shown to increase cellular oxidative stress and further induce tissue damage [50]. Following exposure to chronic mild hypoxia, adaptation is associated with increased vascularity and restoration of metabolic homeostasis. Therefore, adaptation is protective. These results might explain the lack of clinical benefit from hyperoxia as reported by Davies et al. [50].

Adaptation to chronic mild hypoxia may also induce an anti-inflammatory environment. It has been shown that under inflammatory conditions, enhanced T-cell activity can lead to lower oxygen levels and HIF-1 induction [51], which in turn can lead to induction of several genes involved in the glycolytic pathway. Glut-1 (a glucose transporter), hexokinase 2, glucose-6-phosphate isomerase, enolase 1, pyruvate kinase muscle, and lactate dehydrogenase [52, 53] are inducted in T cells. On the other hand, numerous nonhypoxic stimuli including T-cell receptor (TCR) activation [54], nitric oxide, lipopolysaccharides, and infectious microbes can result in HIF-1α up-regulation even under normoxic conditions [55] suggesting that HIF-1 may play multiple roles in leukocytes [51]. It will be important to study the effect of hypoxia as well as EAE using animals with targeted HIF-1 deficiency. Studies by others suggest that HIF-1 modulates T-cell differentiation towards Th17 cytokine-secreting phenotype. Decreased HIF-1α resulted in diminished Th17 development but enhanced T-regulatory cell differentiation protecting mice from autoimmune neuroinflammation [52]. Clambey et al. [53] also showed that HIF-1α induced FoxP3+ Tregs during inflammation. Similarly, we found in our EAE model CD4+ cells were significantly decreased, while the CD4+CD25+FoxP3 Tregs subset was higher in the spinal cords of EAE mice exposed to chronic mild hypoxia when compared to normoxic EAE mice. MOG-specific Th17 responses were found delayed. Thus, exposure to low oxygen appeared to alter key inflammatory pathways important in EAE pathogenesis.

For many years, Th1 cells were deemed responsible for the initiation of autoimmune demyelination. IL-17-secreting cells are also considered a critical effector population in autoimmune demyelinating disease. IL-23 polarizes IL-17-secreting myelin-reactive CD4+ T cells. These cells are highly encephalitogenic upon transfer into naïve syngeneic recipients [1, 56, 57]. Recently, more focus was on Tregs, since it has been shown that there was a resistance to autoimmune demyelination in mice reconstituted with CD4+CD25+FoxP3+ regulatory T cells [28, 58, 59], and Tregs were correlated with the reduced inflammation in EAE [60]. These studies suggested that development of EAE is due to the balance between encephalithogenic and suppressor cells, and adaptation to mild hypoxia favors the induction of suppressor cells. In addition to T cells, chronic exposure to mild hypoxia modulated inflammatory mediators in favor of anti-inflammation. In our studies, we found anti-inflammatory cytokine IL-10 was induced and the inflammatory chemokine CXCL13 was attenuated in sham-immunized animals exposed to hypoxia and during the early phases of hypoxic EAE. CXCL13 has been shown to be detrimental both in EAE and MS [38, 61]. Its levels increase at the onset of MS in a clinically isolated syndrome (CIS) [36, 62], increasing further with exacerbations in RRMS [61] regarded as a biomarker of inflammation in MS [61, 63].

Results in animal models may be useful in human disease. It has been known for some time that a high altitude can promote wound repair [64, 65]. Moderate or intermittent stimulation of deep tissue healing by reduced tissue oxygenation has been shown to promote plasticity. Using mouse models of lymphatic regeneration and inflammatory lymphangiogenesis, HIF-1α was identified as a central regulator [66]. The investigators showed that when HIF-1α was inhibited by small molecule inhibitors (YC-1 and 2-methyoxyestradiol) there was delayed lymphatic repair, decreased vascular endothelial growth factor-C (VEGF-C) expression, reduced numbers of VEGF^+^ cells, and an overall reduction in inflammation. A recent study in human spinal cord injury has shown that daily exposure to intermittent hypoxia enhanced motor function in injured patients [67].

## Conclusions

In conclusion, adaptive angioplasticity is protective and promotes cell survival. Here, we report that the induction of vascular remodeling in MOG-immunized mice significantly delayed the onset of signs and symptoms of clinical disease. Adaptation to chronic hypoxia modulated the immune system by inducing anti-inflammatory mediators and T-regulatory cells. Further study of the adaptive mechanisms that are induced upon exposure to mild oxygen stress may pinpoint new therapeutic targets to promote neuroprotection in chronic CNS inflammation.

## Abbreviations

CD: cluster of differentiation
DAB: diaminobenzidine
DMEM: Dulbecco’s modified Eagle’s medium
EAE: experimental autoimmune encephalomyelitis
FACS: fluorescence-activated cell sorting
FBS: fetal bovine serum
Glut-1: glucose transporter protein-1
HIF-1α: hypoxia-inducible factor-1alpha
IL-10: interleukin 10
MOG: myelin oligodendrocyte glycoprotein
MS: multiple sclerosis
PMNs: Polymorphonuclear leukocytes
SDS: sodium dodecyl sulfate
Th: T helper
